# Critical assessment of staining properties of a new visualization technology: a novel, rapid and powerful immunohistochemical detection approach

**DOI:** 10.1007/s00418-020-01906-5

**Published:** 2020-08-07

**Authors:** M. Seidl, B. Weinhold, L. Jacobsen, O. F. Rasmussen, M. Werner, Konrad Aumann

**Affiliations:** 1 Faculty of Medicine, Medical Center–University of Freiburg, Institute for Surgical Pathology, University of Freiburg, Freiburg i.B., Germany; 2 Agilent Technologies, Glostrup, Denmark

**Keywords:** Immunohistochemistry, Detection system, Tumor pathology, Staining properties

## Abstract

Immunohistochemical staining of tissue sections is a vital technique in pathological diagnostics and theranostics. Several kinds of detection systems are available—each of them with their advantages and disadvantages. Here we present the results of a study assessing a prototype immunohistochemical detection technology (PIDT) for visualization of antigens in tissue sections. Different tumor tissues (*n* = 11) were stained with selected antibodies (*n* = 30) and a subset of these under different fixation conditions. The staining properties were assessed according to six staining quality parameters (signal distribution, intensity, tissue and slide background, acutance, clarity of details, and subcellular morphological details), and the results were compared with those of a well-established detection system (EnVision FLEX). Overall, both detection methods revealed good to optimal results regarding the evaluated parameters even under unfavorable fixation conditions. However, with the prototype detection technology a quicker turnaround time was reached primarily due to shorter primary antibody incubation times. Moreover, PIDT-stained tissues showed higher signal intensity and a uniform signal distribution over the tissue slide, still, with well-preserved tissue morphology and without impairing the gradation of staining intensity of different cell types. In particular, the prototype detection technology performed better in poorly or delayed fixed tissue. In situations where rapid and profound results are in demand, and particularly in the context of a small laboratory setting, this prototype detection technology could be a useful addition to the established detection systems.

**Electronic supplementary material:**

The online version of this article (10.1007/s00418-020-01906-5) contains supplementary material, which is available to authorized users.

## Introduction

Any listing of the virtues of immunohistochemistry would be incomplete if it did not include the visual pleasure derived from examination of this material (…). There is undoubtedly an aesthetic component to the practice of histology (Juan Rosai, in (Dabbs 2013))

Immunohistochemistry (IHC) is a method to localize specific antigens in tissues with the purpose to characterize distinct cell types, mainly for diagnostic and research purposes. Additionally, the field of theranostics—which is composed of the terms therapy and diagnostics and implicates that pathologists serve oncologists and their patients with accurate tissue diagnostics that drive therapies (Dabbs 2013)—would be inconceivable without reliable and rapid immunohistochemical techniques.

Since Coons et al. described the principles of IHC (Coons et al. 1942), the technology has undergone a range of modifications, including enzymatic labeling (Avrameas 1972) before the first application in routinely processed formalin-fixed, paraffin-embedded (FFPE) tissues (Taylor and Burns 1974). Subsequently, modifications have continued to improve IHC, including the development of different amplification methods such as tyramide (Toda et al. 1999) and polymer-based labeling systems (Bisgaard et al. 1993; Heras et al. 1995; Bisgaard and Pluzek 1996; Sabattini et al. 1998). The availability of a wide range of visualization alternatives with different properties is exactly why the selection of an appropriate visualization system is a key element in obtaining optimal staining performance for the given purpose (Giorno 1984; Cordell et al. 1984; Skaland et al. 2010).

The aim of this study was to critically assess a not yet commercially available prototype immunohistochemical detection technology (PIDT) on routinely processed tissue samples. The PIDT builds on an improved tyramide system (Lohse et al. 2014). By use of DAB as a crosslinker in a first deposition step, a very weak primary DAB stain is formed that anchors multiple accessible FITC reporters for a second DAB stain with anti-FITC/HRP. This assures that the stains remain crisp and localized, while still allowing strong amplification with short incubation times. Although there are more steps in the staining procedure, each step, including primary antibody, can be kept at 1–8 min, allowing faster staining on Autostainer Link 48 up to 12–18 slides after which the single robot dispenser becomes the time-limiting factor.

For this purpose, immunohistochemical stainings were performed on tumor and control tissue sections with the novel technology using the well-established EnVision FLEX visualization system (Sabattini et al. 1998) as a reference system. The staining results were evaluated according to a defined scoring system and data were compared statistically.

## Materials and methods

### Study design

Buffered 4% formalin-fixed and paraffin-embedded tissues were used unless otherwise noted. All stains were performed in a routine laboratory setting. All antibodies were ready-to-use and provided by Agilent Technologies. The reactivity of each antibody was tested with appropriate control tissues (e.g. tonsil, appendix, bone marrow, tumors) as used routinely by the Laboratory of Immunohistochemistry of the Department for Pathology, University Medical Center Freiburg, and recommended by Dako’s “Atlas of Stains” (Dako 2012). For all comparisons, the PIDT stains were compared to EnVision FLEX stains, provided by Agilent technologies.

Initially, a phase 1 study was designed to evaluate workflow aspects (incubation times and run-time calculations) and to get a first impression of the staining performance of PIDT using a panel of 30 different primary antibodies reacting with nuclear, cytoplasmic, and membranous antigens. Antibodies used for subtyping epithelial, mesenchymal, and hematological tissues were chosen to cover all major purposes of routine applications. Sections from formalin-fixed, paraffin-embedded tissues of five different tumors were stained with each antibody and both visualization systems (see Suppl. Table 1), totaling 300 sections. After optimization of the protocols using PIDT, a subset of 11 antibodies were selected for phase 2 to further evaluate the general staining characteristics. The staining results of each slide were evaluated and scored as described below.

To test the robustness of PIDT towards different fixation conditions, four samples of fresh tonsil tissue were separately fixed in 4% formalin under defined fixation conditions: under-fixation (4–6 h), standard-fixation (24–48 h), weekend-fixation (48–78 h), over-fixation (7 days), fixation under frozen section conditions (tissue was frozen, thawed, and fixed for 16–18 h), and delayed-fixation (16–18 h fixation after tissue dried out for 2 h). After paraffin embedding, a tissue microarray (TMA) was created containing all four cases with all six different fixation conditions. Each tissue core was 2 mm in diameter. TMA sections were stained using nine different antibodies (BCL6, CD2, CD10, CD20, CD23, CD68, CK-Pan, Ki-67, and p63).

To evaluate the signal quality in poorly fixed tissues, samples from larger resection specimens of mastectomies and pneumectomies with estrogen receptor- or TTF-1-positive breast and lung adenocarcinomas, respectively, were used. These sections were all stained with Ki-67, estrogen receptor, or TTF-1, respectively.

To test if PIDT is able to display distinct differences in signal intensity among different structures within one tissue, the following stainings were performed: Pan-cytokeratin on small-cell lung cancer and CD23 on chronic lymphatic leukemia tissue sections. The stains were evaluated regarding the typically dot-like and—compared to non-neoplastic epithelium—attenuated pan-cytokeratin signal pattern of the tumor cells as well as the attenuated CD23 signal intensity of chronic lymphatic leukemia cells compared to non-neoplastic follicular dendritic cells.

Finally, evaluation of the staining performance in bone marrow biopsies was included in the study as this tissue type represents a special challenge for immunohistochemical staining. Follicular lymphoma-infiltrated bone marrow biopsies were fixed in 4% buffered formalin, subjected to decalcification in a mixture of 10% ethylenediamine tetraacetic acid disodium salt (Serva) and 3.3% tris-(hydroxymethyl) aminomethane (AppliChem) in dd H_2_O at pH of 7.0–7.2 overnight at room temperature and subsequently embedded in paraffin. The biopsies were stained with BCL6, CD10, and CD79a antibodies.

### Pre-analytic treatment

Target retrieval of tissue sections for PIDT staining was done using a single target retrieval solution (TRS), while pretreatment for Envision FLEX staining was performed with either high or low pH TRS according to validated protocols used by the Department for Pathology, University Medical Center Freiburg. De-paraffinization, rehydration, and epitope retrieval were performed with the PT Link, Pre-Treatment Module for Tissue Specimens (Agilent Technologies).

### Staining procedures

All stainings were performed using the Autostainer Link 48 (Agilent Technologies) automated staining platform. For the PIDT, a staining protocol was established (see suppl. Table 2). The appropriate incubation time was optimized for each primary antibody. Validated protocols from the Department for Pathology were used for EnVision FLEX stainings. Appropriate control tissues were included in every run. The stainings with PIDT and EnVision FLEX were performed separately in different runs to be able to assess workflow aspects. Additionally, for each staining run with the PIDT, one slide of consecutively cut sections of tonsil tissue stained with Ki-67 was included for assessment of staining reproducibility.

### Staining evaluation

The specific staining pattern of each antibody used as the basis for the evaluation was defined according to David Dabbs’ “Diagnostic Immunohistochemistry”(Dabbs 2013) and the criteria for optimal staining of the NordiQC (https://www.nordiqc.org). A total of six staining quality parameters to be assessed separately were defined: homogeneity of the specific staining across the tissue, the intensity of the specific signal, tissue background, acutance of the specific signal, clarity of cellular or subcellular morphological details, and slide background. Each parameter was assessed semi-quantitatively using a scale of 3 for optimal, 2 for good, 1 for borderline, and 0 for poor results.

The evaluation was performed by a pathologist who was blinded with respect to which visualization system (PIDT vs EnVision FLEX) was used. A mean value based on the individual scores from the five different tissue sections stained was calculated for each antibody and visualization system. The mean values were used to compare the two visualization systems.

For the reproducibility testing using Ki-67-stained slides, the six parameters were evaluated, and the percentage of nuclear positive cells out of 300 cells was calculated. The mean values of the parameters and the percentage of nuclear positive cells were compared among the staining runs.

### Run time calculation

The duration of each run of the staining was measured from start of barcode reading to completion of final hematoxylin wash, and the number of loaded slides was annotated.

### Statistical analyses

For statistical analyses of data, GraphPad Prism 6 Software (GraphPad Inc.) was used. The scores of the two visualization systems were compared and the statistical significance of differences between the two systems was calculated by use of a paired two-tailed *t* test. When more than two groups were compared a one-way ANOVA test was used. A *p* value of < 0.05 was considered to be statistically significant.

## Results and discussion

In this study, we assessed the properties of a prototype immunohistochemical detection technology (PIDT) by Agilent Technologies in a routine setting. We compared the staining quality and their laboratory workflow impact using different tumor tissues and selected antibodies to compare immunohistochemical staining between the established EnVision FLEX system and PIDT. The staining properties were scored for defined parameters and the results were compared between the two detection systems.

The results show that both techniques provided a high staining quality with only marginal differences in specific staining details. PIDT was found to provide a more homogeneous distribution of staining intensity across the tissue and, in bone marrow biopsies, a signal enhancement, which led to an increase in staining quality.

### Short primary antibody incubation times lead to significantly reduced assay run time especially with low numbers of slides

Optimization of the protocol for PIDT demonstrated that very short incubation times for the primary antibody were required: only 1 min incubation time for 12 of the primary antibodies and 2 min for nine of the antibodies. The longest incubation time was 8 min among the 30 antibodies. A considerable advantage of PIDT was the short incubation time of the primary antibody of 1–2 min for most antibodies resulting in a slide number-dependent run time decrease of 2.75× (up to five slides) and 1.8× (up to ten slides). A total of 76 staining runs were conducted, 53 runs with PIDT and 23 runs with EnVision FLEX. The average total staining run time on Autostainer Link 48 was 58 min (range 30–109 min) for the PIDT and 111 min (range 89–149 min) for EnVision FLEX with statistically significant difference (*p* < 0.0001). The difference in total staining run time depended on the number of slides included in the respective staining runs where the assay run time is shown for the following groups of slides: low volume (1–6 slides), medium volume (7–12 slides), and high volume (13–19 slides). With an increasing number of slides, the mean staining time per run increased significantly with PIDT (low: 40 min, medium: 57 min, and high: 77 min; *p* < 0.0001), whereas the staining run time with EnVision FLEX changed only to a minor extent (low: 102 min, medium: 109 min, and high: 120 min; *p* = 0.0518). Comparison of slide run time within each group showed that the staining run times were significantly lower with PIDT compared to EnVision FLEX (each *p* < 0.0001 in pairwise comparison).

To test the reproducibility of the staining, each run included a section of tonsil tissue that was stained with Ki-67 antibody and evaluated for the proportion of cells showing nuclear staining. The mean percentages of nuclear Ki-67-positive cells were compared resulting in a high reproducibility of the stainings without statistically significant differences between the single runs and a standard deviation of only 1.7% regarding the percentage of Ki-67-positive cells.

### PIDT displays superior signal homogeneity and comparable signal intensity to EnVision FLEX

**Fig. 1:**
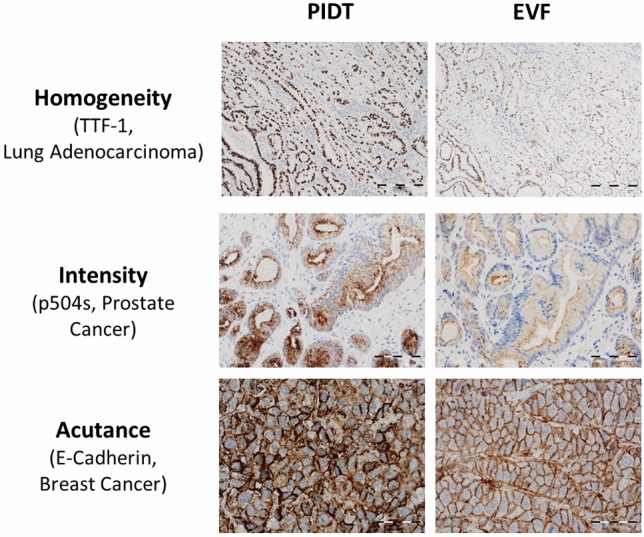
Representative examples of staining results for selected antibodies in tumor tissues. Bar indicates 200, 100 and 50 µm in the first, second and third line, respectively

To evaluate the staining performance of PIDT, tumor tissue slides were stained with both detection systems using eleven different antibodies. Five different cases were used for each antibody. The results were compared between the two detection systems. Overall, staining with PIDT resulted in a good to optimal homogeneity of signal distribution across the tissue sections. However, in a few cases stained with EnVision FLEX a variance in homogeneity was seen with strong to moderate positive cells near the edge, whereas cells in the center were unstained or weakly stained. In contrast, tissue sections stained with PIDT showed a more uniform distributed signal without local variation across the whole section. Comparing the results regarding the homogeneity of signal distribution, a statistical significant difference between the two detection systems was found (Fig. 1).

**Fig. 2:**
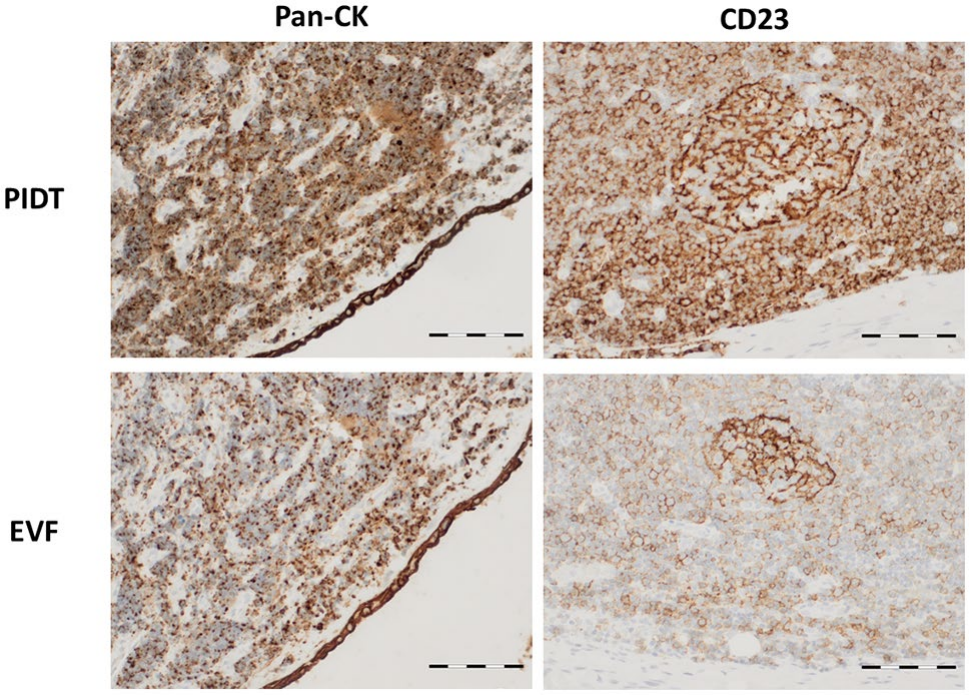
Examples of PIDT and EnVision FLEX staining results of distinctions in signal intensity of different cell populations within a tumor, exemplarily shown is dot-like pan-cytokeratin staining in cells of a small cell lung cancer compared to stronger staining of the local epithelium and stronger CD23 staining of dendritic cells compared to infiltrating CLL cells in a lymph node. Bar indicates 100 µm

Generally, PIDT provided good to optimal results regarding staining intensity. The difference in staining intensity was, however, not significant compared to EnVision FLEX. It should be noted that despite increased intensity by PIDT, refined distinctions in signal intensity of different cell populations within one tumor type was displayed very well (Fig. 2).

The strong signal intensity in combination with the homogenous signal distribution over the tissue section led to a uniform staining result. This property may contribute to more consistent results of quantitative evaluation of immunohistochemical slides, e.g. in case of defining the intrinsic subtypes of breast cancer. Furthermore, computer-assisted evaluation of immunohistochemical stained tumor tissue by image processing may profit from the strong and homogenous signal of PIDT.

Another point is the yet unknown cause behind the higher signal homogeneity in the PIDT stains. There was no difference between the primary antibodies used. Furthermore, regarding the different quality assessment parameters there is no significant difference between the target retrieval solutions (high vs low ph) used for EnVision FLEX stains, especially not for homogeneity (data not shown). Moreover, the incubation time for the primary antibodies in the PIDT protocol is fairly shorter. Therefore, our findings suggest an improved binding or reduced dissociation of the primary antibody in the PIDT stains. This could be due to the quickly introduced DAB/FITC precipitates coating the primary antibody and preventing dissociation.

The overall strong signal intensity of PIDT staining led to the question if refined distinctions in staining intensity in some tumor entities are still detectable. Accordingly, tissue sections of small-cell lung cancer (SCLC) and cases of lymph nodes infiltrated by chronic lymphatic leukemia/small lymphocytic lymphoma (CLL/SLL) were stained with Pan-cytokeratin or CD23 antibodies, respectively. Both the attenuated cytokeratin positivity of SCLC cells and the enhanced signal intensity of CD23-positive follicular dendritic cells compared to the CLL/SLL tumor cells could be reliably detected. These results show that the high intensity of PIDT-staining did not impair the detection of signal graduation of different cell types within the tested samples.

### PIDT and EnVision FLEX show comparable very low background, EnVision FLEX with higher scores for acutance and morphological details

Both detection systems showed nearly no slide background and extraordinary low level of tissue background staining without statistically significant differences. Interestingly, despite the higher intensity of PIDT staining only a small increase of background staining could be measured which was not significantly different compared to EnVision FLEX indicating that the specific signal was enhanced by PIDT without enhancing background signal. For PIDT, the acutance was good, however, EnVision FLEX reached a very high score resulting in a statistically significant difference between both detection systems. Finally, tissue slides stained with PIDT showed clearly recognizable, morphological details, however, a better performance of EnVision FLEX could be measured with a statistically significant difference.

### PIDT with increased signal intensity and homogeneity of bone marrow biopsies

**Fig. 3:**
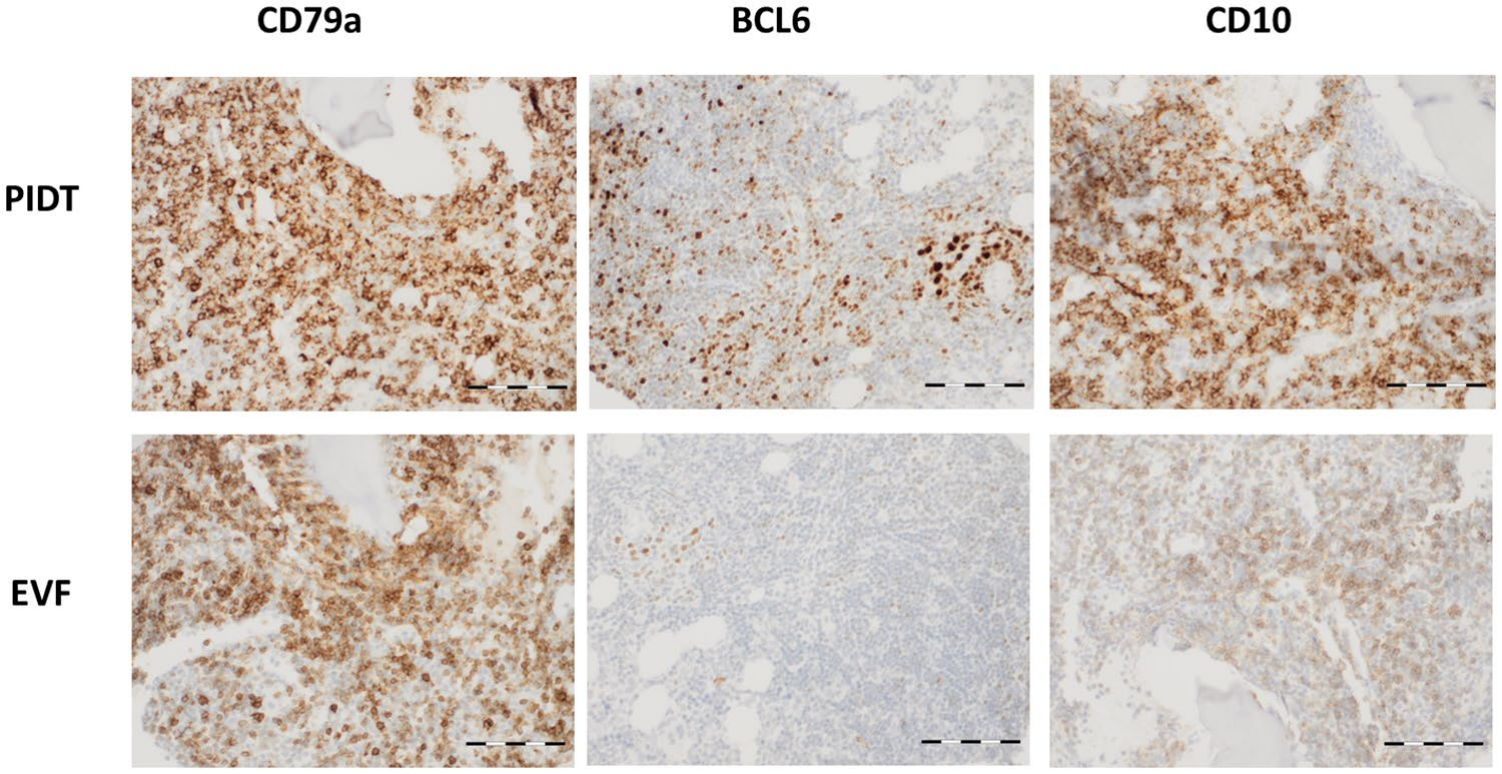
Examples of PIDT and EnVision FLEX staining results of bone marrow biopsies with infiltration of follicular lymphoma. Bar indicates 100 µm

Assessment of bone marrow staining was included as this tissue represents a particular challenge for good immunohistochemical staining quality due to special requirements for pretreatment to ensure the correct balance between antigen retrieval and tissue preservation. Bone marrow biopsies of five follicular lymphoma-infiltrated cases were stained in parallel with both detection systems and each case with BCL6, CD10, and CD79a antibodies. The slides were scored as described above and scoring results were compared between the detection systems. Overall, both PIDT and EnVision FLEX stainings of bone marrow biopsies showed clear and specific signals with a well-balanced intensity in combination with well-preserved tissue morphology. PIDT staining showed increased signal intensity and homogeneity compared to EnVision FLEX for CD10- and BCL6 antibodies while acutance of CD79a-staining was slightly impaired (see Fig. 3).

### PIDT and EnVision FLEX are comparably robust to fixation differences

Proper immunohistochemical staining of tissue is very challenging for a detection system when tissue sections are sub-optimally fixed. To analyze the robustness of PIDT and EnVision FLEX towards fixation differences some procedure variations were imitated. For this reason, we created a tissue micro array (TMA) with cores of tonsil tissue with over-fixed, under-fixed, and delayed-fixed tonsil tissue cores as well as tonsil tissue fixed after freezing and thawing (analog frozen section). Immunohistochemical stainings with BCL6, CD2, CD10, CD20, CD23, CD68, CK AE1/3, Ki-67, and p63 were conducted. The stainings were evaluated for staining quality parameters as described above. Indeed, frozen sections yielded the lowest staining scores. However, none of the unfavorable fixation conditions impaired the staining quality of either detection system to the extent that staining results were unacceptable for evaluation. Moreover, there was no statistically significant difference regarding the staining results of PIDT and EnVision FLEX stained tissues.

Overall, both PIDT and EnVision FLEX revealed good to optimal results and were highly able to compensate different unfavorable fixation conditions with average staining scores between 2.4 and 3.0. The only statistically significant difference was seen for signal homogeneity over the tissue section, where PIDT showed better values, and for the acutance of the staining, where EnVision FLEX yielded a sharper signal.

Both detection systems yielded good to optimal staining results regarding the individual fixation conditions. Statistically significant differences could be measured regarding the homogenous distribution of the signal over the tissue section when the tissue was fixed too shortly, over the weekend, and under standard conditions.

**Fig. 4:**
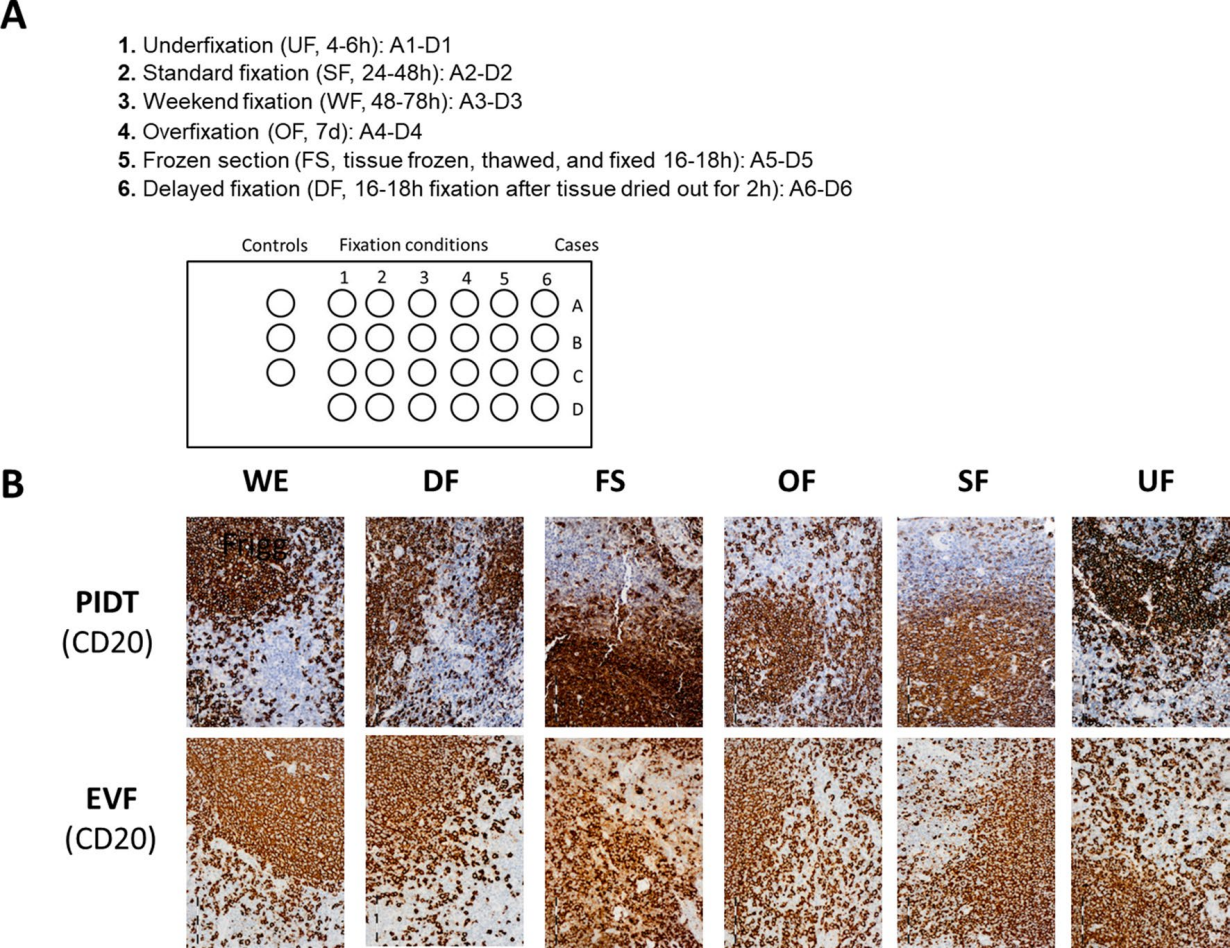
Example of staining results of a TMA containing spots with different fixed tonsil tissue stained for CD20 with PIDT and EnVision FLEX. **a** Description of the TMA; **b** Staining results. Bar indicates 100 µm

Signal quality in tissue fixed analog to the frozen-section setting was impaired regarding acutance and morphological details. Still, good staining scores were assessed without statistically significant differences between PIDT and EnVision FLEX. Delayed fixation had no adverse effect on immunohistochemical staining quality regarding all parameters as shown by good to optimal staining scores with EnVision FLEX yielding significantly better results for intensity and acutance (Fig. 4).

**Fig. 5:**
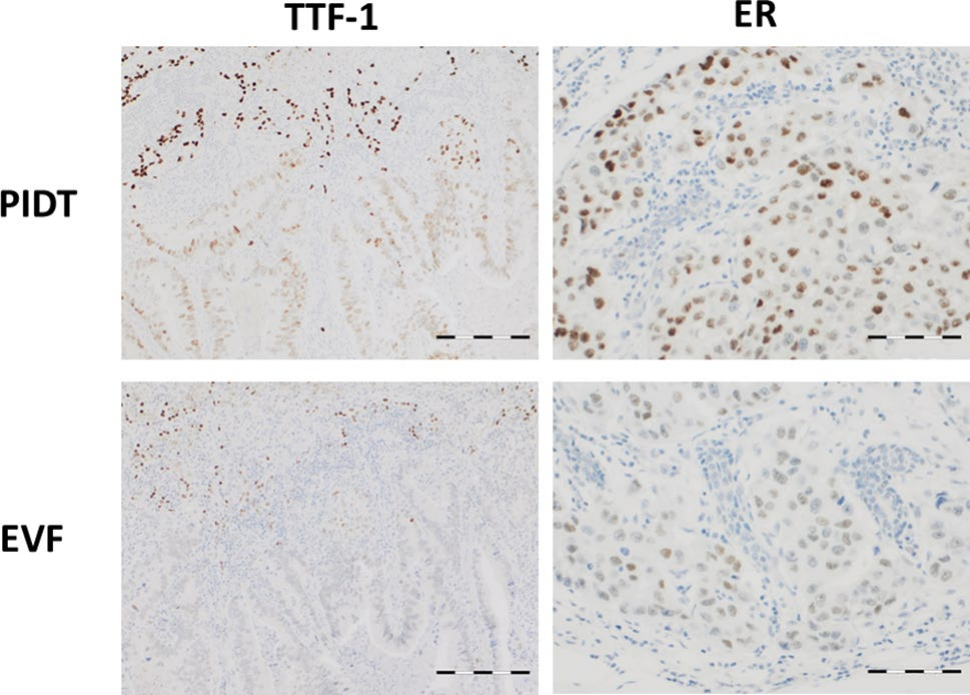
Examples for PIDT and EnVision FLEX staining results of poorly fixed tissue (lung adenocarcinoma and breast cancer stained with TTF-1 or estrogen receptor, respectively). Bar indicates 100 µm

To test if the quality of immunohistochemical staining is affected by differently fixed areas within large tissues, tissue sections of resection specimens of adenocarcinoma of mastectomies and pneumectomies with differently fixed areas were stained with antibodies to Ki-67, estrogen receptor, or with TTF-1, respectively. The staining results were evaluated as described above and compared between PIDT and EnVision FLEX. Again, both detection systems showed good to optimal staining results with slightly better values for PIDT but without statistically significant differences between the two detection systems. However, one lung adenocarcinoma was TTF-1-negative using EnVision FLEX but was weakly positive in the PIDT staining (Fig. 5).

In summary, both detection systems PIDT and EnVision FLEX are of comparable high quality regarding the staining results. PIDT is a powerful technology in particular due to its high intensity and homogeneity of the signal distribution over the tissue section especially in bone marrow biopsies. From a laboratory perspective, PIDT enables short run times, which can be particularly useful in pre-therapeutic situations where the challenge is to provide rapid and profound results. Furthermore, laboratories with rather low immunohistochemical throughput would benefit from PIDT as the strength of this technology becomes particularly beneficial when a smaller number of slides per run is stained.

## Electronic supplementary material

Below is the link to the electronic supplementary material.

Supplementary file1 (DOCX 14 kb)

Supplementary file2 (PDF 270 kb)
