# Supplementary material for: Critical assessment of staining properties of a new visualization technology: a novel, rapid and powerful immunohistochemical detection approach
Source: Histochem Cell Biol. 2020 Aug 7;154(6):663–9. doi: 10.1007/s00418-020-01906-5 (PMC7723932; doi:10.1007/s00418-020-01906-5)
Supplement: Supplementary file 1 — Supplementary file1 (DOCX 14 kb) [file 418_2020_1906_MOESM1_ESM.docx]

Suppl. Table 1: Incubation times of the primary antibodies according to the detection system. PIDT= prototype immunohistochemical detection technology, EVF = EnVision FLEX detection system.

| **Antigen** | **Clone** | **Incubation Times (Minutes)** | | |
| --- | --- | --- | --- | --- |
|  |  | **PIDT** | **EVF** | |
| AMACR | 13H4 | 2 | 20 |  |
| ASMA | 1A4 | 2 | 20 |  |
| BCL6 | PG-B6p | 8 | 20 |  |
| CEA | II-7 | 2 | 20 |  |
| CD2 | AB75 | 2 | 20 |  |
| CD10 | 56C6 | 2 | 30 |  |
| CD20 | L26 | 1 | 10 |  |
| CD23 | SP23 | 2 | 20 |  |
| CD34 | QBEnd 10 | 2 | 30 |  |
| CD56 | 123C3 | 5 | 40 |  |
| CD68 | PG-M1 | 1 | 20 |  |
| CD79a | JCP117 | 3 | 20 |  |
| CD99 | 12E7 | 2 | 10 |  |
| CDX2 | DAK-CDX2 | 3 | 20 |  |
| CK5/6 | D5/16 B4 | 5 | 30 |  |
| CK7 | OV-TL12/30 | 3 | 15 |  |
| CK20 | Ks20.8 | 1 | 20 |  |
| Chromogranin | poly | 1 | 20 |  |
| CyclinD1 | SP4 | 1 | 40 |  |
| Cytokeratin | AE1/AE3 | 1 | 20 |  |
| E-Cadherin | NCH-38 | 6 | 20 |  |
| Epithelial Antigen | Ber-EP4 | 1 | 20 |  |
| Estrogen Receptor | EP1 | 3 | 20 |  |
| Kappa | poly | 1 | 1 |  |
| Lambda | poly | 1 | 1 |  |
| Ki-67 | MIB-1 | 2 | 20 |  |
| Melanosome | HMB45 | 3 | 20 |  |
| S100 | poly | 1 | 20 |  |
| TTF-1 | 8G7G3/1 | 6 | 10 |  |
| Vimentin | V9 | 1 | 20 |  |
|  |  |  |  |  |
|  | Mean | 2.5 | 19.9 |  |
|  | Median | 2.0 | 20.0 |  |
|  | Max | 8.0 | 40.0 |  |
|  | Min | 1.0 | 1.0 |  |

Suppl. Table 2: PDIT Staining Procedure

| **Step** | **Category** | **Reagent** | **Incubation Time** |
| --- | --- | --- | --- |
| 1 | Rinse | Buffer |  |
| 2 | Endogenous Enzyme Block | PDIT Peroxidase-Blocking Reagent | 3 min |
| 3 | Rinse | Buffer |  |
| 4 | Primary Antibody |  | as appropriate |
| 5 | Rinse | Buffer |  |
| 6 | Secondary Reagent | PDIT Dual Link Reagent | 5 min |
| 7 | Rinse | Buffer |  |
| 8 | Rinse | Buffer |  |
| 9 | Auxillary Reagent | PDIT Auxillary Reagent (Working Solution) |  |
| 10 | Rinse | Buffer |  |
| 11 | Rinse | Buffer |  |
| 12 | Tertiary Reagent | PDIT Tertiary Antibdy Reagent | 3 min |
| 13 | Rinse | Buffer |  |
| 14 | Substrate-Chromogen | PDIT DAB Substrate (Working Solution) | 3 min |
| 15 | Rinse | Buffer |  |
| 16 | Counterstain | Hematoxylin | 3 min |
| 17 | Rinse | DI Water |  |
| 18 | Rinse | Buffer |  |
| 19 | Rinse | DI Water |  |
