# Supplementary material for: Critical assessment of staining properties of a new visualization technology: a novel, rapid and powerful immunohistochemical detection approach
Source: Histochem Cell Biol. 2020 Aug 7;154(6):663–9. doi: 10.1007/s00418-020-01906-5 (PMC7723932; doi:10.1007/s00418-020-01906-5)
Supplement: Supplementary file 2 — Supplementary file2 (PDF 270 kb) [file 418_2020_1906_MOESM2_ESM.pdf]

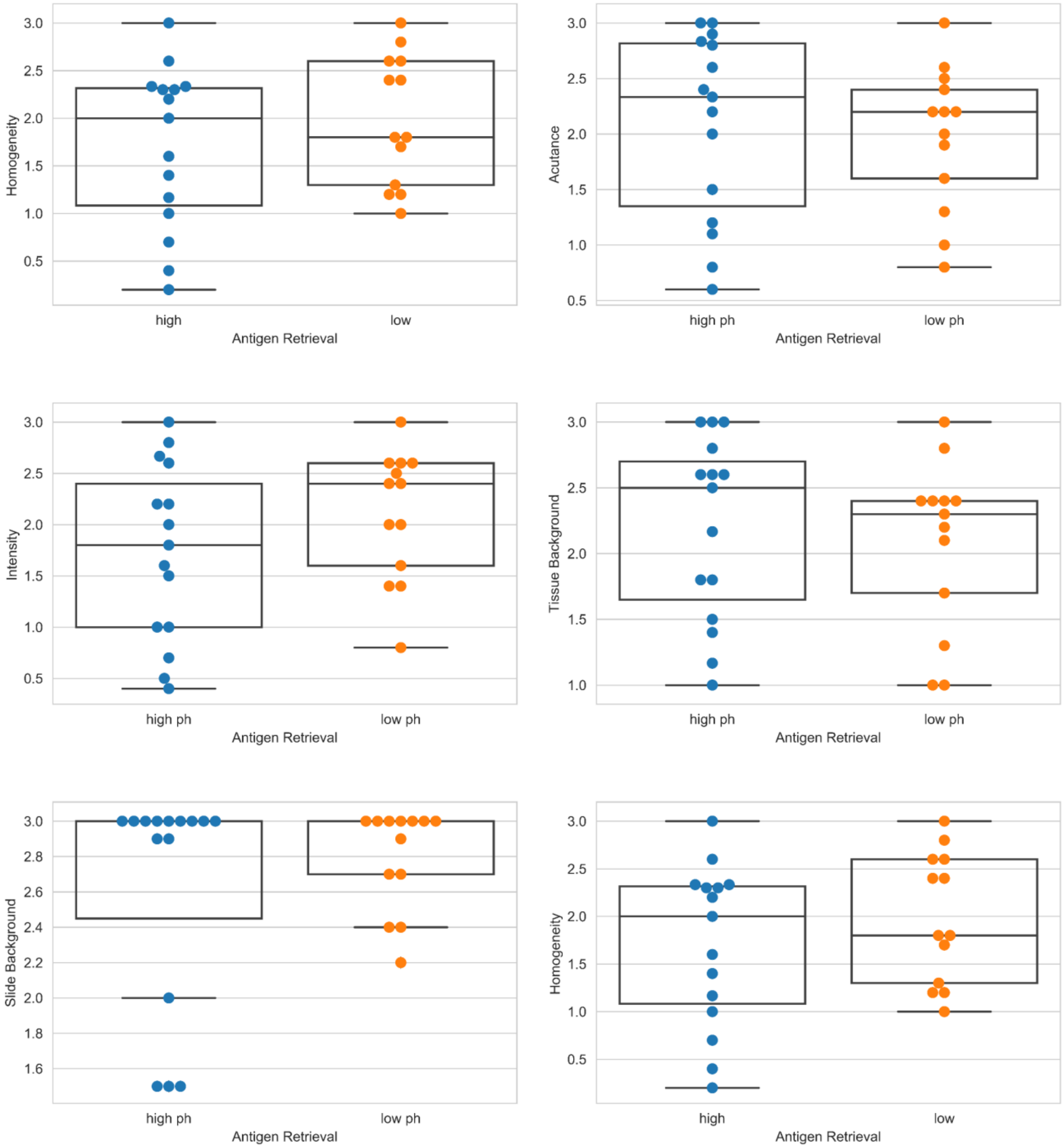

Overview of different quality parameters measured from high vs. low ph antigen retrievals. Each dot represents a different antibody. Boxes indicate the standard deviation together with the median line. As most of the values display a normal distribution but some do not, correlation between antigen retrievals (high and low ph) and quality assessment values was tested using the correlation coefficient according to Pearson (normal distributed, tests for linear correlation) and Spearman (rank correlation coefficient). None of the correlation coefficients showed significant correlations between the different quality assessment parameters and the antigen retrieval. Correlation coefficients and quality assessment parameters are provided in the crosstabs of the next page.

	AR (1high_0low)	Homogen	Slide Bkg	Tissue Bkg	Intensity	Acutance	Details
AR (1high_0low)__CORR	1	-0.20864836	-0.00984323	0.15578322	-0.18668247	0.12442075	0.0710585
AR (1high_0low)__p-Value	0	0.2866455	0.96035201	0.42859679	0.34150729	0.52816165	0.71935721
AR (1high_0low)__number	28	28	28	28	28	28	28
Homogen__CORR	-0.20864836	1	0.6663099	0.57974603	0.79071106	0.5293312	0.48015932
Homogen__p-Value	0.2866455	0	0.00010843	0.00122345	5.53E-07	0.00377345	0.00971043
Homogen__number	28	28	28	28	28	28	28
Slide Bkg__CORR	-0.00984323	0.6663099	1	0.63769512	0.67079085	0.58957918	0.52821373
Slide Bkg__p-Value	0.96035201	0.00010843	0	0.00026198	9.36E-05	0.00096123	0.00386143
Slide Bkg__number	28	28	28	28	28	28	28
Tissue Bkg__CORR	0.15578322	0.57974603	0.63769512	1	0.71782928	0.70027644	0.74243022
Tissue Bkg__p-Value	0.42859679	0.00122345	0.00026198	0	1.71E-05	3.34E-05	6.09E-06
Tissue Bkg__number	28	28	28	28	28	28	28
Intensity__CORR	-0.18668247	0.79071106	0.67079085	0.71782928	1	0.63713895	0.66992049
Intensity__p-Value	0.34150729	5.53E-07	9.36E-05	1.71E-05	4.05E-201	0.00026628	9.64E-05
Intensity__number	28	28	28	28	28	28	28
Acutance__CORR	0.12442075	0.5293312	0.58957918	0.70027644	0.63713895	1	0.92550866
Acutance__p-Value	0.52816165	0.00377345	0.00096123	3.34E-05	0.00026628	4.05E-201	1.81E-12
Acutance__number	28	28	28	28	28	28	28
Details__CORR	0.0710585	0.48015932	0.52821373	0.74243022	0.66992049	0.92550866	1
Details__p-Value	0.71935721	0.00971043	0.00386143	6.09E-06	9.64E-05	1.81E-12	0
Details__number	28	28	28	28	28	28	28

Spearman rank correlation coefficient

	AR (1high_0low)	Homogen	Slide Bkg	Tissue Bkg	Intensity	Acutance	Details
AR (1high_0low)__CORR	1	-0.18430977	-0.17437672	0.09102388	-0.2420341	0.07357762	0.07595749
AR (1high_0low)__p-Value	0	0.34778808	0.37482416	0.64504843	0.21464882	0.70982549	0.70085971
AR (1high_0low)__number	28	28	28	28	28	28	28
Homogen__CORR	-0.18430977	1	0.77802183	0.67080958	0.84629584	0.61095538	0.57965289
Homogen__p-Value	0.34778808	0	1.10E-06	9.36E-05	1.40E-08	0.00055361	0.0012262
Homogen__number	28	28	28	28	28	28	28
Slide Bkg__CORR	-0.17437672	0.77802183	1	0.68161176	0.74081414	0.6980641	0.68265018
Slide Bkg__p-Value	0.37482416	1.10E-06	0	6.50E-05	6.54E-06	3.63E-05	6.28E-05
Slide Bkg__number	28	28	28	28	28	28	28
Tissue Bkg__CORR	0.09102388	0.67080958	0.68161176	1	0.77913186	0.7609401	0.82372658
Tissue Bkg__p-Value	0.64504843	9.36E-05	6.50E-05	0	1.04E-06	2.59E-06	7.25E-08
Tissue Bkg__number	28	28	28	28	28	28	28
Intensity__CORR	-0.2420341	0.84629584	0.74081414	0.77913186	1	0.68552221	0.69964821
Intensity__p-Value	0.21464882	1.40E-08	6.54E-06	1.04E-06	0	5.68E-05	3.42E-05
Intensity__number	28	28	28	28	28	28	28
Acutance__CORR	0.07357762	0.61095538	0.6980641	0.7609401	0.68552221	1	0.94017306
Acutance__p-Value	0.70982549	0.00055361	3.63E-05	2.59E-06	5.68E-05	0	1.14E-13
Acutance__number	28	28	28	28	28	28	28
Details__CORR	0.07595749	0.57965289	0.68265018	0.82372658	0.69964821	0.94017306	1
Details__p-Value	0.70085971	0.0012262	6.28E-05	7.25E-08	3.42E-05	1.14E-13	0
Details__number	28	28	28	28	28	28	28

Pearson correlation coefficient
